# A new visualization and conceptualization of categorical longitudinal development: measurement invariance and change

**DOI:** 10.3389/fpsyg.2015.00289

**Published:** 2015-03-27

**Authors:** Jan Boom

**Affiliations:** Developmental Psychology, Utrecht UniversityUtrecht, Netherlands

**Keywords:** measurement invariance, strategy development, overlapping waves, latent growth modeling, item response theory

## Abstract

The Overlapping Waves Model (OWM) is a metaphor introduced by Siegler (1996) to illustrate a typical sequence of increasing and decreasing use of strategies during development. Going beyond metaphor, a new model synthesized from Latent Growth Modeling (LGM) and Item Response Theory (IRT) will be presented to analyze such categorical longitudinal data. Use of strategies can be scored as a variable with only a few ordinal categories. IRT provides the means to relate the usage of strategies to position on an underlying developmental dimension. LGM allows to model movement of individuals along this dimension, acknowledging individual differences both in starting point and in speed of progress. Measuring and modeling such strategy development requires that at each time point the same categories are used, in the sense that item difficulties must remain invariant over time. Whether, discrimination can be relaxed is still an issue. The problem that had to be solved was disentangling the between-person-individual differences from real intra-individual developmental differences. Figures with polytomous or multi-category Item Characteristic Curves (ICC's) resemble the OWM in many respects. However, such figures are usually taken to represent inter-individual differences, whereas the OWM usually represents development (so intra-individual differences), and we cannot have both at the same time. The solution came from creating a framework with ability differences on one axis and the effect of time on another axis, resulting in a 3-D model. These (orthogonal) dimensions make it possible to adequately conceptualize measurement invariance in this complex context. As the result is difficult to conceptualize without extensive visualization, special 3-D figures will be used to illustrate and a dynamic (rotatable and scalable) version will be made available as Computable Document Format object (Mathematica). The model was successfully applied in several microgenetic studies.

## Introduction

Measurement invariance (MI) is mostly considered in the context of differences between subpopulations (inter-individually), however, measurement invariance is also important in a longitudinal context. It might be unfair if an instrument does not measure the same constructs for all subpopulations. However, in a longitudinal study, if we compare the same sample (ignoring attrition for the moment) with itself on different occasions, the issue is perhaps not fairness but whether the study is effective in being able to identify progress.

The case I want to focus on concerns strategy development. With development individuals might move from using one strategy to another. Such strategies might be qualitatively different and hierarchically ordered. Let's suppose they are and that the following assumptions about the underlying structure of the data hold to a sufficient degree.

The first assumption is that the strategies can be ordered in terms of advancedness. Higher numbered strategies are better (although it might be difficult to define exactly in what sense) such that each next strategy may become attractive once the presently used one is sufficiently mastered. The second assumption is that participants use only one strategy at a time. This assumption does not necessarily contradict what has become received view: that there is—and has to be—a broad variation in strategies from which—like in evolution—the best strategies are chosen (Siegler, 1996). However, even such a view implies that there are different strategies. Thus, while participants may have several strategies at their disposal, the assumption is that one problem is solved, in the end, by using only one strategy. The next moment, or the next problem, may involve the use of other strategies, so in that sense “use” still might be a mixture. The third assumption is closely related to the previous two and holds that there is a single underlying dimension which represents advancedness of these strategies. This implies that the strategies will map on an ability scale with strategies as markers along this scale and defining the scale. The intervals between strategies along the scale need not be regular but the scale is assumed to be one-dimensional only. Using a strategy can now be scored as an ordinal variable with few categories and longitudinal development as a vector of such scores per participant. These assumptions are compatible with many classical developmental theories: developmental scales, stage theories, skills theory, and hierarchical complexity theory.

I have proposed a formal statistical model to analyze such data by connecting group level trends to an underlying developmental dimension valid on the individual level. Let me explain first why this is has been a problem so far in developmental psychology before returning to MI and details.

**Figure 1:**
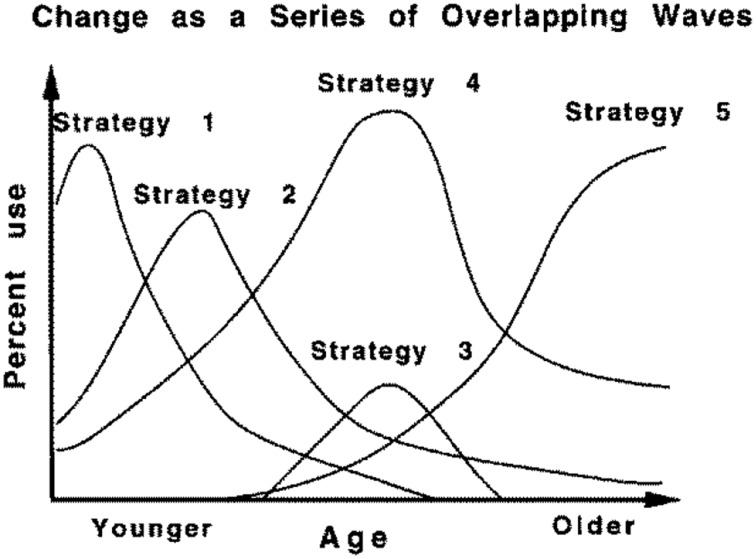
**Overlapping waves depiction of cognitive development**. From “Emerging Minds,” by Siegler (1996).

**Figure 2:**
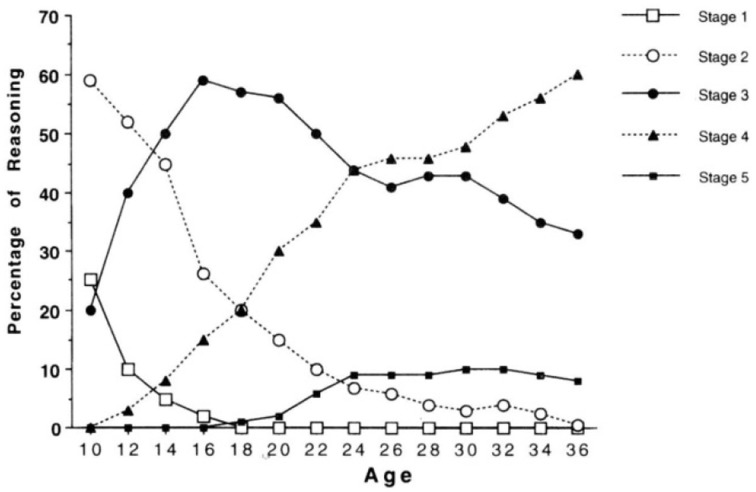
**Percentage use of stages of moral reasoning**. Data from Colby et al. (1983).

The Overlapping Waves Model (OWM) was introduced by Siegler (1996) as a metaphor to illustrate the typical pattern for many cognitive tasks of a sequence of increasing and decreasing use of strategies, or rules as he called them, during *individual* development (Figure 1). Such a pattern might apply, for example, to children learning to multiply numbers below 10: Strategy 1 might refer to incorrect approaches such as guessing; Strategy 2 might refer to finger counting; A more advanced strategy is repeated addition; The most advanced strategy in this example is retrieval from memory. Compare this model to results from a famous longitudinal study by Colby et al. (1983) on stage-wise moral development, in which they reported increasing and decreasing use of five stages of moral development for the group level (Figure 2).

A fundamental problem, that has plagued developmental theorizing since long, is that it is difficult to infer the shape of development from group results. Figure 2 refers to actual empirical data, but trends are only valid on the group level; whereas, Figure 1 suggests being valid on the individual level, but does not directly reflect empirical data (it's just hypothetical). On the one hand, because the shape of non-linear trends need not be comparable between group and individual data, referring to group data as in Figure 2 will not do as support for claims about typical *individual* trajectories. On the other hand, actual individual data (trajectories), as e.g., presented abundantly in Siegler (1996) and Colby et al. (1983), have not been used to formally confirm trends as in Figure 1, possibly because the overwhelming individual variation. Of course, there are likely to be constraints and relationships between the individual and the group level, but a formal model of the exact nature of these relationships was lacking so far.

Going beyond metaphor, I developed a new conceptualization synthesized from Latent Growth Modeling (LGM) and Item Response Theory (IRT) to understand such categorical longitudinal data. The model itself is not new, because Muthén explored such models extensively (Muthén, 1996; Muthén and Asparouhov, 2002, 2013), but the application to strategy development is entirely new. IRT provides the means to relate the likelihood of use of particular strategies to a position on an underlying developmental dimension. LGM allows modeling movement of individuals along this dimension, acknowledging individual differences both in starting point and in speed of progress (and more).

Measurement Invariance must hold in such models in the sense that strategies themselves do not change with time or age. What is supposed to change is the use, or the propensity to use them. MI might be violated if a new factor has become influential over time, the result would be that we cannot find progression developmentally. Whether discrimination can be relaxed is an issue to be discussed below. I will first introduce IRT and LGM.

## Item response theory modeling

With the assumptions in place Item Response Theory (IRT) provides the means to relate the use of strategies to an underlying ability.

**Figure 3:**
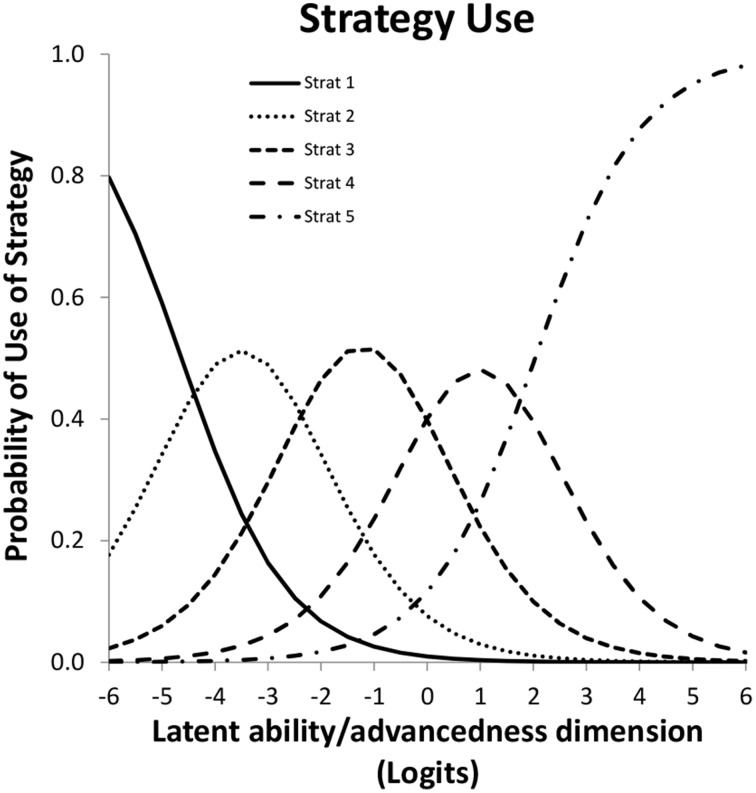
**Item characteristic curves for a five-category polytomous item**.

Suppose responses of participants are scored as representing the use of one of a few strategies, suppose furthermore -following IRT modeling- that the likelihood of using one of the strategies depends on a single latent variable by a mathematical function known as the item response function. The Partial Credit Model is particularly useful to model responses that are ordered as a series of steps that must be mastered in sequence (Millsap, 2010). The latent variable normally represents inter-individual differences in ability, which in this case translates to being more or less advanced in terms of strategy use as is illustrated in Figure 3. Figures are based on a micro-genetic study (Van der Ven et al., 2012) and just used to illustrate here. The multiplication strategies examples given earlier are from this study.

The X-axis of Figure 3 can be thought of as representing strategy ability differences between participants (as person characteristic). In that case a position more to the right represents a higher ability, more to the left represents less ability. On the Y-axis is the likelihood of using the particular strategy. For example, likelihood of using strategy-5 is higher for persons with high strategy ability, while strategy-1-use diminishes rapidly with increasing ability.

Alternatively, the X-axis can be used to represent characteristics of the strategy; e.g., the peak of each of the middle curves gives the most typical ability value for that strategy, but it is also clear there is considerable overlap between strategies.

The result is a latent strategy ability scale that can be used to represent inter-individual ability differences and strategy advancedness. The position along this strategy scale is nonlinearly and probabilistically related to the use of the various strategies. The attractiveness of the transformation of the categorical scores to this unbounded continuous interval scale is that it opens up the possibility to use all kinds of regression techniques.

However, one of the key ideas of this paper is that the X-axis can also be used to represent intra-individual development, as in Siegler's Overlapping Waves Model. In other words: also development over time can be conceptualized and visualized as a shift to right in Figure 3. The result in terms of expected strategy use can be quite complex to describe because it depends on the starting point and the growth rate of the particular subject. Only the first and the last strategy have a consistent change pattern, the use of all other strategies goes up and down. The profile of shapes depends on properties of the item and may be different for different items. For a Partial Credit model version the basic shape (steepness of the curves) is fixed. Although scaling of X-axis is arbitrary, location (to the right or left) can vary between items, and applies to the whole set of curves for an item. Height of the curves, or area's beneath it, which can also be expressed as distance between crossings of curves, may be different within or between items.

Basic principles of relevant IRT modeling, and some alternative models, are reviewed by de Ayala (2009) and Embretson and Reise (2000), more details on polytomous item response models can be found in Ostini and Nering (2006), and more on categorical data-analysis in general in Agresti (2002).

## Latent variable growth curve modeling

Whereas, IRT provided the means to relate the use of strategies to an underlying dimension, development of individuals along this underlying dimension can be modeled by means of Latent variable Growth curve Modeling (LGM). LGM is a powerful and flexible technique, which can be used to model longitudinal development (Bollen and Curran, 2006; Duncan et al., 2006). A linear LGM, for example, presupposes a steady increase or decrease in the target variable over a small number of equally spaced measurement occasions for each person. The increase is assumed to be linear, with an intercept and a slope parameter describing a trajectory for each individual. These intercepts and slopes are assumed to be different for each respondent and normally distributed in the population with unknown mean and variation. In the usual continuous case, the observed scores for an individual participant will depart from his or her best fitting straight line, and it is assumed that these residuals are normally distributed in the population with zero mean and certain variance for each measurement occasion; moreover, these residuals are not correlated over measurements occasions. However, in the present case, where we combine the LGM with an IRT model, the role of residuals can be treated in other ways too. Because this has consequences for the issue of MI I will return to the role of residuals in a moment.

Concluding: a linear LGM might be suitable to model increasing strategy development over measurement occasions, and, if the model holds, every participant's trajectory can be represented by a straight line, as will be shown shortly.

## Three dimensional overlapping waves model

The Overlapping Waves model of Figure 1 was originally presented to visualize development, whereas the polytomous item response model of Figure 3 is more commonly used to depict individual differences. With a three-dimensional version of the Overlapping Waves model both uses can be combined.

**Figure 4:**
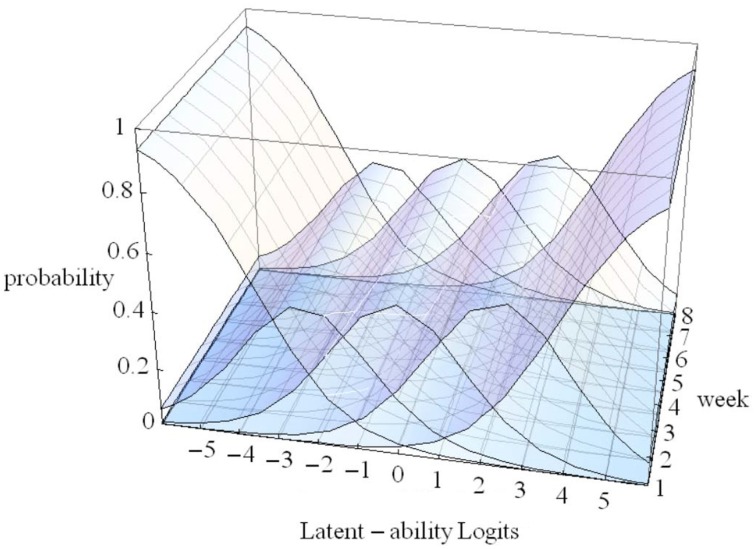
**3D-Overlapping waves model (see text)**.

**Figure 5:**
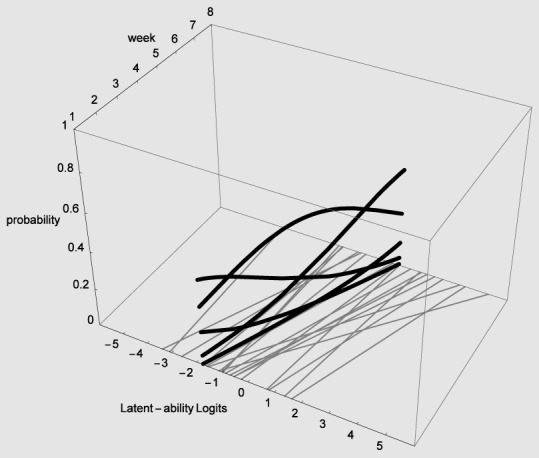
**LGM trajectories (thin gray lines) and snapshot of Item Characteristic Curves for one participant (heavy black lines)**.

In Figure 4 the X-axis refers to individual differences, the Y-axis to time (measurement occasions = 8 weeks in our empirical example), and the Z-axis refers to probability of using one of five strategies. The floor is a two dimensional plane on which a growth curve model can be placed as illustrated in Figure 5.

**Figure 6:**
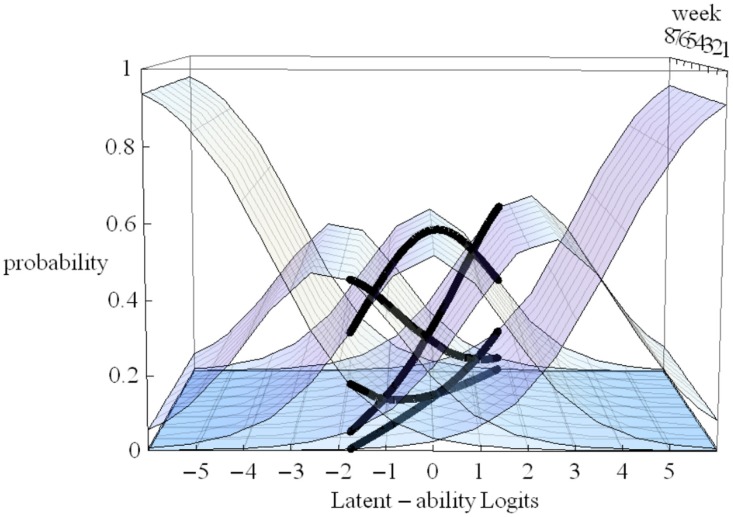
**3D-Overlapping waves model with item characteristic curves for one participant as illustration**.

In Figure 5 estimated (idealized) individual trajectories of development in strategy use are shown on the floor plane for a subsample of 20 participants. For one individual, as illustration, the implied category boundaries are also depicted in the Z-plane. The degree of curvature is limited, which makes sense, because it represents only a modest increase on the strategy maturity scale as in Figure 3. Figures 4, 5 are combined in Figure 6 to illustrate that each individual curve from Figure 5 follows the surface of the waves in Figure 4.

The more growth, in Figure 6, the more the set of curves for a particular individual turns away from an orientation parallel to the week axis, and the more curvature (in the heavy black lines) will result. No growth, as e.g., for the case with the line most to the left on the floor of Figure 5, would result in straight heavy black lines for each of the 5 strategies when projected on the surfaces. But also a different starting point (different intercept in the growth model part) can lead to completely different curvatures: imagine the set of curves being shifted along the difficulty dimension.

## Measurement invariance revisited

Since we are interested in development of using strategies over time some assumptions had to be made. In principle an assumption cannot be tested (not directly at least). Scoring observations or verbal material etc. needs to be done first and this often implies a considerable amount of preprocessing of the raw data. Categories have to be defined in advance, so that judges can apply these to the observations. The categories must be defined such that they are ordered in terms of difficulty. Age of respondents whose responses are to be assigned to the categories cannot and may not play a role at all in the definition of the categories. Nor should time or measurement occasion play a role in the definition of the categories. Therefore, if we relate these categories to an underlying ability this relation must remain strictly invariant over measurement occasions.

Specifying the relation between categories and underlying ability can be done in many ways, but always involves the difference between the latent ability score and thresholds. A threshold τ_*j*_ is the value on the scale where the likelihood for being assigned category changes from being greater for *j* to being greater for *j* + 1 (so around *p* = 0.5) and represent what in IRT parlance would be the difficulty of the item.

For a weighted least-squares (WLS) estimator with Probit link and Theta parametrization the Item Characteristic Curves (ICC) is specified as follows: Let *U*_*i*_ be a categorical indicator for a latent ability factor *f* with categories *j* = 0, 1, 2,…, *J*-1, for item *i* = 1, 2,… *I*, and measurement occasion *t* = 1, 2,… *T.*

(1) $$\begin{array}{l} P_{itj}\left(f\right)\;=P\left(U_{it}=j|f\right)\\ \;=\Phi \left(\frac{\tau_{itj}-\lambda_{it}f}{\sqrt{\theta_{it}}}\right)-\Phi \left(\frac{\tau_{itj-1}-\lambda_{it}f}{\sqrt{\theta_{it}}}\right) \end{array}$$

Where Φ is the standard normal distribution function, τ_*j*_ is the threshold for category *j*, λ is the factor loading, θ is the residual variance. For the first category the second Φ term is zero, for the last category the first Φ term is 1. Note, however, that Mplus offers not only WLS estimators but also maximum likelihood (ML) estimators, not only Probit links but also Logit links, and also a Delta parametrization (see Muthén, 2010 for an overview), and for each case the ICC's are differently specified. Figure 3 is an example of a ML Logit ICC, however, apart from scaling differences the Figure would be almost the same as the one obtained from formula 1.

Regarding difficulty; with more items and more measurements occasions the thresholds τ_*ij*_ (for each separate category) are to remain invariant over occasions, but may be different over items *i*. Steps may be defined as s_*j*_ = τ_*ij*_ − τ_*ij*−1_ and restricted, or not, to be equal between items.

Regarding discrimination; dividing by the standard deviation of the residuals θ_*it*_ in formula 1 allows introducing differences in discriminations over items and occasions. With ML estimators this is difficult to achieve and discrimination differences are not implemented in Mplus for ML. Being able to allow differences (with WLS) in relative discrimination between items is however an attractive option that can improve fit. Whether, allowing the residual variance θ_*it*_ to be variant over measurements occasions, as is advocated by Muthén and Asparouhov (2002), is a good idea has to be seen. It will undoubtedly improve fit but interpretation might be more difficult. In Figures 3, 5 for some occasions the Figure will in that case be broader or slimmer which is detrimental to the intended general applicability of the model: As outlined above the intention is to be able to handle large individual differences in ability with this model.

## Example: stepwise understanding randomness

The general model has been applied to several data sets now (e.g., Van der Ven et al., 2012). The pilot study presented below is just meant as an illustration.

Children's understanding of randomness was studied using a microgenetic design in three age cohorts (Grade 1, 3, and 5) of different primary schools in rural parts of the Netherlands. During 5 weeks four probability-related questions about a marble tilt box (see Metz, 1998) were administered weekly to 75 children. A box 30 cm wide and 40 cm long, with edges 5 cm high was mounted on a support of 5 cm high, affixed to the bottom, such that it could be tilted from one side to the other such that marbles would roll from one side to the other. Initially all marbles were lying in row on the lower side: 5 white on the left and 5 green on the right. Questions were asked before, during, or tilting of the box: e.g., “How will the marbles end up on the other side?”; “Can you be sure”; “What happened?” (After trying it out themselves); “What if we did tilt the box a 100 times?”; “Can the original distribution of the marbles (5 and 5 neatly separated) ever occur again?” Answers given by each child were coded using 4 possible categories (based on Metz, 1998). Developmental progress was presumed to go from: (1) No understanding at all to (2) determinism which is denial of chance element (they have to go back to their places), to (3) unpredictability (you never know, they just roll), to (4) recognizing some degree of long term predictability (returning is possible but unlikely). Teaching about randomness is not a part of curriculum in the Netherlands. Participants were not given feedback by the test administrators, but were able to see, of course, the outcomes when the task was eventually played each week. This resulted in a 75 (participants) by 16 (variables = 4 question-sets by 4 weeks) raw data-matrix with codes one to four.

## Results

**Figure 7:**
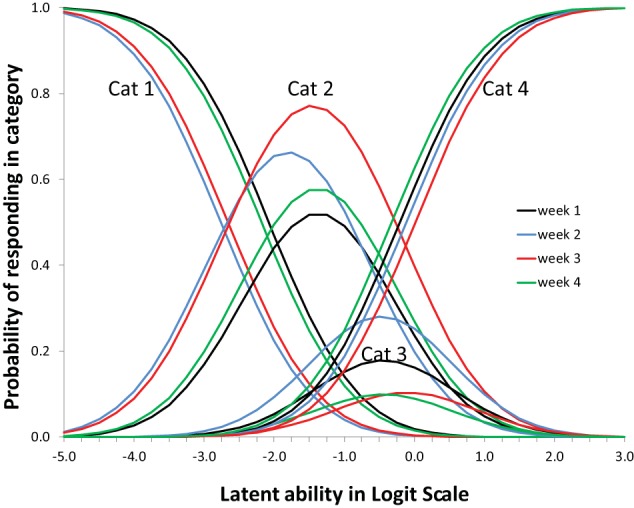
**Item profiles for four topic/question sets**. On the x-axis is the latent ability/advancedness scale, as explained earlier.

**Figure 8:**
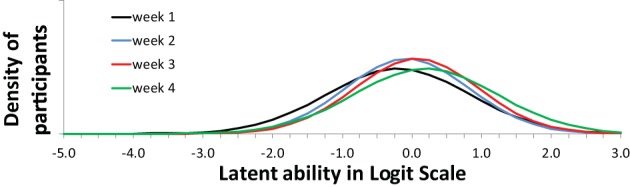
**Model Implied (expected) week trends of use of categories (levels of understanding)**.

Figure 7 shows that the four items (= topic/question-set) had different profiles. These shapes of the item profiles are fixed over the 4 weeks (to achieve measurement invariance). On the x-axis is the difficulty of the item (to use the IRT parlance) which in this case reflects whether the particular question tends to elicit more advanced or more simplistic responses. Note that children can be placed on the same x-axis scale: see Figure 8.

Figure 8 shows the expected changes in the use of the categories (levels of understanding), over weeks, for each item (topic/question-set), separately. The scale on the x-axis corresponds exactly to Figure 7. The actual abilities, in this case, cover only small part of the scale (the scale is centered around zero because the average is arbitrarily set to zero).

Increase over weeks (the slope in the growth model part) was significant. Nevertheless, seen from a substantive viewpoint, the result are not all good. Category three has very low occurrence (never dominant) and might be better removed from the coding scheme. The range of respondent abilities is not corresponding to the item categories very well. No substantive conclusions from this illustration can be drawn. However, seen from a modeling and analysis viewpoint results are good and interesting. Mplus 7.2 was used to estimate and fit all models (Muthén and Muthén, 1998-2012). Fit for the WLS Probit version with DELTA parameterization was acceptable with an RMSEA of 0.075; CFI of 0.911; TLI of 0.930. More options for analysis were tried out (e.g., MLR-Logit, WLS-THETA) and all converged to the same kind of Figures (as in Figures 7, 8). The analysis demonstrates that with a rather small sample already interesting results are possible and weaknesses in the data or coding scheme are revealed without fail.

**Figure 9:**
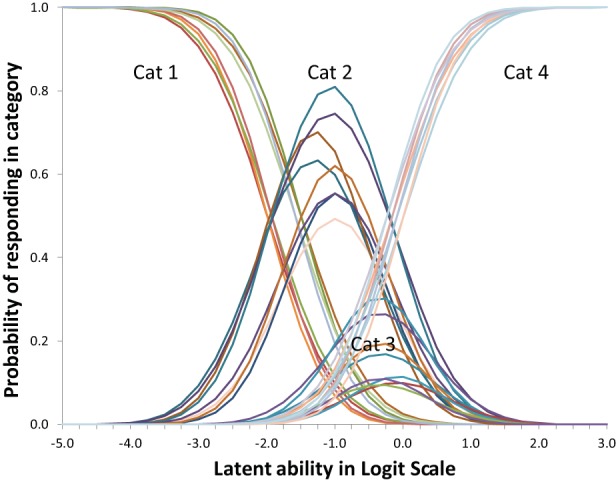
**Item profiles for four topic/question sets, while allowing for some measurement differences between weeks**. On the x-axis is the latent ability/advancedness scale, as explained earlier.

Regarding measurement invariance it might be argued that there might be systematic differences over 4 weeks, e.g., due to slightly different testing conditions. Using the scaling option in the Mplus DELTA parameterization we allowed scaling differences between measurement points. The resulting scaling factors were 1 (as anchor) for set 1 and 1069, 0.890, and 1.093, respectively for sets 2–4. Fit for this WLS Probit version with DELTA parameterization was almost the same with an RMSEA of 0.074; CFI of 0.914; TLI of 0.932. The scaling option in the DELTA parameterization gave slightly better results than the option to relax the discrimination (requiring the THETA parameterization) as mentioned earlier, but has the same kind of effect: introducing mild between measurement differences by just stretching or contracting the scale a bit. The result is Figure 9 with four times as many, and more cluttered, lines than Figure 7.

## Discussion

A new 3D Overlapping Waves model is presented, based on a combination of Latent variable Growth curve Modeling (LGM) and Item Response Theory (IRT) modeling. The statistical principles used are long established and sound. It is a formal model for conceptualizing strategy development which throws new light on the issue of variability and measurement invariance in development. It is also an empirically testable model which might be helpful in longitudinal studies in which the responding changes fundamentally over development or experience.

All advantages of LGM apply. Predictors can be added to the LGM part, it is also possible to test nonlinear growth, or add more growers to the model. All advantages of IRT modeling also apply. The new part is: that what is normally the end result (estimated individual scores or group indicators thereof) now is -in an additional step- transformed in a set of thresholds and these can be visualized as a set of curves (with strong shape constraints). More hypotheses concerning the relationship between strategies can be tested by specifying equality constraints between thresholds. Also the relationship between items (e.g., more or less difficult ones) can be further investigated and tested. IRT analyses are often based on much larger datasets; large item banks, and focused on item selection for a test. The present application of IRT is different and more experience with dealing with complex models with relatively few participants is needed.

The raw data may appear incredibly complex and variable, but, as shown, it may still be the case that the data are generated by relatively simple linear growth for each person reflecting an underlying dimension of development. Of course, in actual practice there will always be violations of the assumptions, for all kinds of reasons, but if there is a consistent pattern over individuals to a sufficient degree, such an underlying dimension is plausible. The analysis can be done with commercially available software and is not difficult to conduct, although it requires some conceptual work and spatial imagination. The model has important theoretical implications!

Regarding measurement invariance over weeks, the illustrative example showed that relaxing the strict measurement invariance, by allowing some overall scaling differences, did not lead to serious improvement in fit, but since all the threshold are different the Figure is more difficult to understand and also difficult to compare to a more restricted model. Although more studies are needed before final recommendations can be given, a more parsimonious model seems preferable.

### Conflict of interest statement

The author declares that the research was conducted in the absence of any commercial or financial relationships that could be construed as a potential conflict of interest.
